# Establishment and verification of prognostic model and ceRNA network analysis for colorectal cancer liver metastasis

**DOI:** 10.1186/s12920-023-01523-w

**Published:** 2023-05-09

**Authors:** Xuan Zhang, Tao Wu, Jinmei Zhou, Xiaoqiong Chen, Chao Dong, Zhangyou Guo, Renfang Yang, Rui Liang, Qing Feng, Ruixi Hu, Yunfeng Li, Rong Ding

**Affiliations:** 1 Department of Colorectal Surgery, Yunnan Cancer Hospital, The Third Affiliated Hospital of Kunming Medical University, Kunming, China; 2 Department of Minimally Invasive Intervention, Yunnan Cancer Hospital, The Third Affiliated Hospital of Kunming Medical University, Xishan District, No. 519, Kunzhou Road, Kunming, 650118 China; 3 Department of Oncology, Yunnan Cancer Hospital, The Third Affiliated Hospital of Kunming Medical University, Kunming, China

**Keywords:** Colorectal cancer liver metastases, Prognostic model, ceRNA network, Tumor microenvironment, Bioinformatics, TCGA

## Abstract

**Objects:**

Colorectal cancer (CRC) is one of the most common cancers in the world. Approximately two-thirds of patients with CRC will develop colorectal cancer liver metastases (CRLM) at some point in time. In this study, we aimed to construct a prognostic model of CRLM and its competing endogenous RNA (ceRNA) network.

**Methods:**

RNA-seq of CRC, CRLM and normal samples were obtained from The Cancer Genome Atlas (TCGA) and Gene Expression Omnibus database. Limma was used to obtain differential expression genes (DEGs) between CRLM and CRC from sequencing data and GSE22834, and Gene Ontology and Kyoto Encyclopedia of Genes and Genomes functional enrichment analyses were performed, respectively. Univariate Cox regression analysis and lasso Cox regression models were performed to screen prognostic gene features and construct prognostic models. Functional enrichment, estimation of stromal and immune cells in malignant tumor tissues using expression data (ESTIMATE) algorithm, single-sample gene set enrichment analysis, and ceRNA network construction were applied to explore potential mechanisms.

**Results:**

An 8-gene prognostic model was constructed by screening 112 DEGs from TCGA and GSE22834. CRC patients in the TCGA and GSE29621 cohorts were stratified into either a high-risk group or a low-risk group. Patients with CRC in the high-risk group had a significantly poorer prognosis compared to in the low-risk group. The risk score was identified as an independent predictor of prognosis. Functional analysis revealed that the risk score was closly correlated with various immune cells and immune-related signaling pathways. And a prognostic gene-associated ceRNA network was constructed that obtained 3 prognosis gene, 14 microRNAs (miRNAs) and 7 long noncoding RNAs (lncRNAs).

**Conclusions:**

In conclusion, a prognostic model for CRLM identification was proposed, which could independently identify high-risk patients with low survival, suggesting a relationship between local immune status and prognosis of CRLM. Moreover, the key prognostic genes-related ceRNA network were established for the CRC investigation. Based on the differentially expressed genes between CRLM and CRC, the prognosis model of CRC patients was constructed.

**Supplementary Information:**

The online version contains supplementary material available at 10.1186/s12920-023-01523-w.

## Introduction

Colorectal cancer (CRC) is the third most common cancer with a high metastasis and recurrence rate [1]. In the past decade, the diagnosis and treatment of CRC have been greatly improved. However, distant metastases, especially liver metastases lead to the poor prognosis and high fatality rate in CRC patients [2]. Approximately 14–25% of CRC patients have simultaneous liver metastasis, while 20–33% of patients have metachronous liver metastasis. Radical resection of metastasis is still the first choice for colorectal cancer liver metastases (CRLM), but only 10%-20% of patients are suitable for radical resection [3]. Therefore, it is necessary to better understand the pathogenesis and provide more effective treatment for CRLM, and early detection of liver metastases is urgent for the prognosis and survival of CRC patients.

The tumor microenvironment (TME) is composed of many different and interacting cell populations, which is closely related to the prognosis and response to treatment. Many factors produced by immune, stromal, or malignant cells, remodel TME and the adaptive immune response culminating in liver metastasis [4–6]. Activation of the Wnt signaling pathway and migration of granulocytes might take a vital role in CRLM [7]. The NOTCH1 signaling could drive metastasis through transforming growth factor (TGF) β-dependent neutrophil recruitment in TME [8]. The abnormal aggregation of immune cells, like tumor associated macrophages (TAMs) [9], regulatory T cells (Tregs) [10] and natural killer cells (NK cells) [11] in TME significantly affected the prognosis and metastasis of CRC.

Competing endogenous RNA (ceRNA) regulated target mRNA expression at the post-transcriptional level through competing for miRNAs binding sites. As a bridge, ceRNA connects the function of coding mRNAs with non-coding RNA [11–13]. Many studies have indicated that ceRNA was involved in pathogenesis and metastasis of CRC in vitro and in vivo experiments [14–17]. MIR4435-2HG is mainly involved in tumorigenesis and metastasis through miR-206/YAP1 axis [16]. LncRNA UICLM promotes CRLM by acting as a ceRNA for microRNA-215 to regulate ZEB2 expression [17]. Therefore, it is highlighting to investigate the roles of ceRNA in pathogenesis and prognosis of CRLM.

In addition, several studies have constructed ceRNA networks closely related to the pathogenesis of CRC through bioinformatics methods [18, 19].The MIR4435-2HG- and ELFN1-AS1-associated ceRNA subnetworks affected and regulated the expression of the seven target genes and were found to be related to prognosis and tumor-infiltrating immune cell types [18]. KCNQ1OT1 ceRNA network could be involved in regulation of TME and survival of CRC patients [19]. However, ceRNA networks and prognostic models of CRC based on liver metastasis-associated genes are lacking.

This theory focuses on the potential prognostic genes which were asscoiated with the identifications for CRLM samples that are different from the primary CRC case and are accompanied with worse prognosis. The differentially expressed genes (DEGs) between the CRC and CRLM samples were selected to construct a prognostic risk models. And meanwhile, the potential mechanism relevant to the key prognostic genes were evaluated by identifying the associated tumor immune microenvironment components and constructing the targeted ceRNA network based on the TCGA-CRC cohorts. Our study aims to construct a prognosis model for CRC patients based on differentially expressed genes between CRLM and CRC.

## Materials and methods

### Clinical samples collection

A total of 10 CRC samples with LM and 10 CRC samples without LM from The Third Affiliated Hospital of Kunming Medical University were enrolled in the study. Seven pairs of samples were subjected to qRT-PCR, while three pairs of samples were subjected to transcriptome sequencing. The sample information was shown in Additional file 1: Table S1. Written informed consent was obtained from all participating patients prior to enrollment into the study. Study protocols were approved by the Ethics Committee of The Third Affiliated Hospital of Kunming Medical University, based on the ethical principles for medical research involving human subjects of the Helsinki Declaration.

### Data source

Gene expression and clinical data were obtained from The Cancer Genome Atlas Program-colon adenocarcinoma (TCGA-COAD) and TCGA-rectum adenocarcinoma (TCGA-READ), which contain 51 normal colorectal and 622 CRC samples (611 of which have survival data). The GSE22834, GSE29621, GSE12945 and GSE72718 datasets were obtained from the Gene Expression Omnibus (GEO) database, GSE22834 included a total of 31 CRC samples and 32 CRLM samples, GSE29621 included a total of 65 CRLM samples and all with survival data, and GSE72718 included a total of 19 CRC samples and 9 CRLM samples. GSE12945 included a total of 62 CRC samples with survival data. In addition, 3 CRC samples and 3 CRLM samples were from our own sequencing.

### Analysis of differentially expressed genes (DEGs)

The differentially expressed analysis in the sequencing data and GSE22834 was performed to screen DEGs between CRC and CRLM with |log2 fold change(FC)|> 1 and *p* < 0.05 as did differentially expressed miRNAs (DEmiRNAs) and differentially expressed lncRNAs (DElncRNAs) in CRC and normal samples using the- “limma”-package in R [20].

### Gene ontology (GO) and Kyoto encyclopedia of genes and genomes (KEGG) function enrichment analysis

The DEGs between the CRC and CRLM samples in sequencing data were were overlapped with that in GSE22834 data to conduct the Gene ontology (GO) and Kyoto Encyclopedia of Genes and Genomes (KEGG) function enrichment analysis [21–23], which was performed using the “clusterProfiler” package in R.

### Construction and verification of a prognostic model

First, the TCGA cohort was randomly divided into a training set (n = 415) and a test set (n = 207) according to a 7:3 ratio. Univariate Cox regression analysis of the overlapped DEGs was performed to screen genes with *p* < 0.05 which were significantly associated with survival of patients in the TCGA-training cohorts. Least absolute shrinkage and selection operator (LASSO) regression analysis was further utilized to select the prognosis genes for the risk model construction using the “Lasso” package in R. Next, the risk score for each patient in TCGA-training set was calculated according to the following formula: Risk score = Σ Coef j × Exp j, where Coef j and Exp j represent coefficients and relative gene expression of these prognosis genes, respectively. Following the median risk score was considered as the cut-off value, the individuals from TCGA-training set were divided into high-risk and low-risk groups. And meanwhile, the different risk groups of TCGA-testing set, GES29621 and GSE12945 set were generated in the same way, respectively. Kaplan–Meier (K–M) survival analysis was utilized to test whether risk score was associated with prognosis. To evaluate the predictive accuracy of the risk score model, the time-dependent receiver operating characteristic (ROC) curve analysis in 1-, 3-, and 5 years was arranged using the “survivalROC” package, where the area under the curve (AUC) values was positively correlated with predictive accuracy. Moreover, clinical informations among four cohorts was extracted, and scatter diagram of risk score and survival states as well as clinicopathological heatmaps of prognostic genes expression differences in age, sex, grade, TMN staging, and survival status were drawn to explore the clinical correlation of prognostic genes.

### Construction and validation of a nomogram

To provide clinicians with a quantitative method for predicting the probability of survival at 1-, 3-, and 5 years in CRC patients, we developed a nomogram [21] that integrates various clinical risk factors in a prognostic model. The nomogram was screened for prognostic factors by univariate and multivariate Cox regression analysis. The calibration curves [22] of the nomogram were generated by plotting the predicted probabilities of the nomograms against the observed ratios, where the best prediction results occurred when the slope was close to 1. Simultaneously, the ROC and decision curve analysis (DCA) curves were plotted for the prognostic accuracy and clinical utilize of the nomogram.

### Gene set enrichment analysis (GSEA)

To investigate the enriched biological processes and signaling pathways that differ between CRC samples of the high- and low-risk group, and the functional mechanisms of most significantly and non-significantly expressed differential genes, the Gene set enrichment analysis (GSEA) was performed by using “clusterProfiler”- package in R.

### Immuno-infiltration analysis

Single-sample gene set enrichment analysis (ssGSEA) was performed using the “GSVA”package in R to derive enrichment scores for each immune-related term to assess the level of infiltration of 28 immune cell species. The spearman method was used to determine the correlation coefficients.

### Competing endogenous RNA (CeRNA) network construction

First, we intersected the “prognostic genes” with the different expression (DE) mRNA in TCGA to obtain the “key prognostic genes”. Then Miranda software was used to predict the target miRNAs of the key prognostic genes, and next the predicted miRNAs were intersected with the DEmiRNAs in TCGA to obtain “key miRNAs”. Then Miranda software was used to predict the target lncRNAs of the key miRNAs, and then the predicted lncRNAs were intersected with the DElncRNAs in TCGA to obtain “key lncRNAs”. The competing endogenous RNA (ceRNA) network was constructed based on these key lncRNAs, key miRNAs, key prognostic genes and visualized using Cytoscape 3.6. Spearman correlation analysis was conducted for the correlation between key RNAs which were involved in the ceRNA network and 28 immune infiltration cells. The K-M survival analysis was used to evaluate the association of key RNAs expression and prognosis in TCGA-CRC cohorts.

## Quantitative real-time PCR

Total RNA was isolated from CRC samples with/without LM using RNA extract (Servicebio, Guangzhou, China) and reverse transcribed using SureScript-First-strand-cDNA-synthesis-kit (Servicebio, Guangzhou, China). PCR conditions were forty cycles at 95 °C for 1 min, followed by 95 °C for 20 s, 55 °C for 20 s, and 72 °C for 30 s. The relative expression of genes is calculated by the 2^−△△Ct^ method. The sequences of the qRT-PCR primers are listed in Additional file 2: Table S2.

## Results

### Identifification of DEGs between CRC and CRLM

**Fig. 1:**
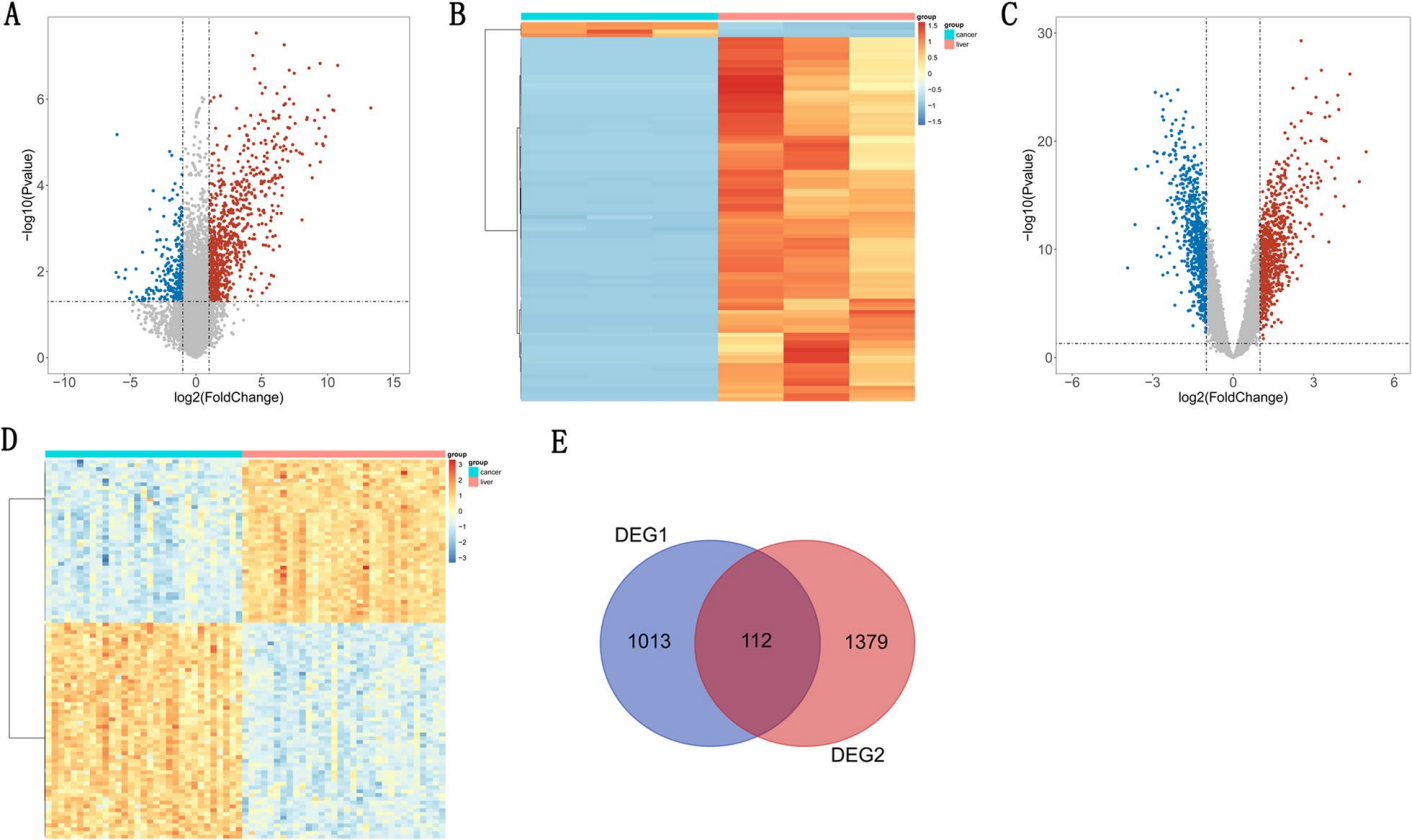
DEGs of CRC and CRLM samples in transcriptome sequence data and GSE22834. **A** A total of 1125 DEGs in transcriptome sequence data. **B** Heatmap of top 100 DEGs between CRC and CRLM in transcriptome sequence data. **C** A total 1491 DEGs in GSE22834. **D** Heatmap of top 100 DEGs between CRC and CRLM in transcriptome sequence data. **D** Heatmap of top 100 DEGs between CRC and CRLM in GSE22834. **E** Co-DEGs between transcriptome sequence data and GSE22834

As shown in Fig. 1A, a total of 1125 DEGs between CRC and CRLM samples were detected in our sequencing data, which including 885 up-regulated and 270 down-regulated DEGS, and the heatmap of top 100 DEGs were shown in Fig. 1B. In the GSE22834 we found a total 1491 DEGs, including 805 up-regulated and 686 down-regulated DEGs (Fig. 1C), and the heatmap of top 100 DEGs were illustrated in Fig. 1D. After that, 112 co-DEGs between sequencing data and the GSE22834 dataset were detected and displayed by Venn graph (Fig. 1E).

**Fig. 2:**
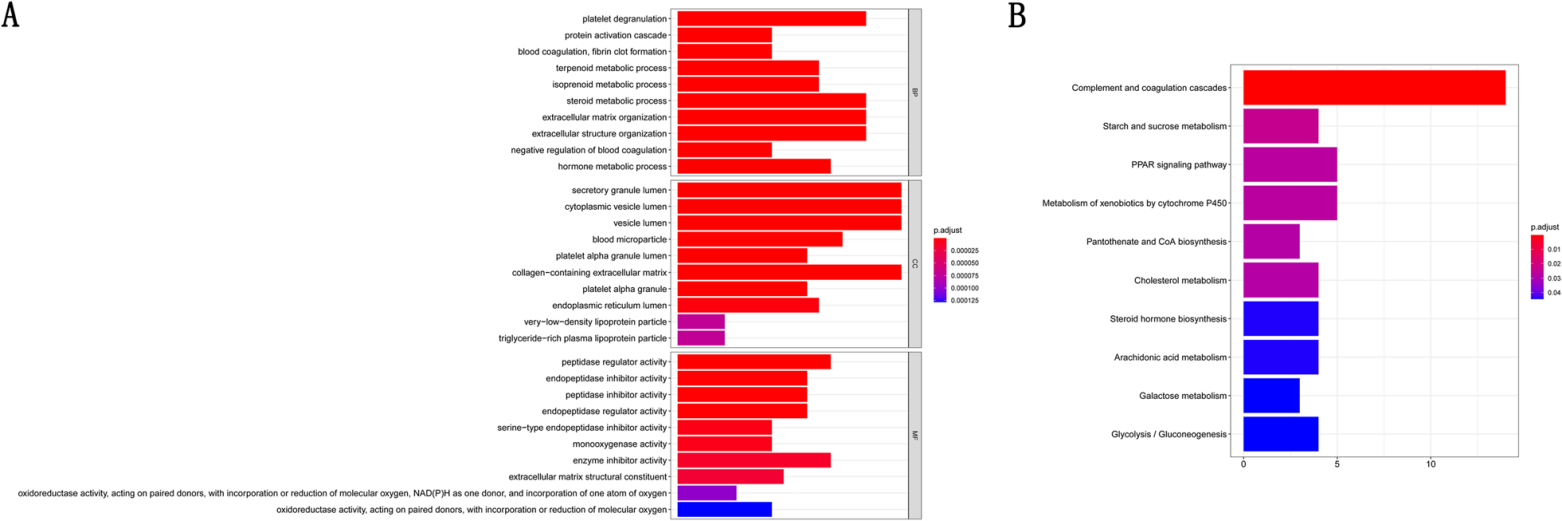
The function of 112 DEGs. **A** Enriched GO enrichment analysis of 112 DEGs. **B** Enriched KEGG pathways of 112 DEGs

In order to found the function of 112 co-DEGs, GO and KEGG pathways enrichment analysis were performed. They were enriched in 264 GO biological processes (BPs), 21 GO cellular component (CCs), 44 GO molecular functions (MFs), and 14 KEGG pathways (Fig. 2A, B).

### Identification of prognosis-related DEGs and establishment of eight gene prognostic model

**Fig. 3:**
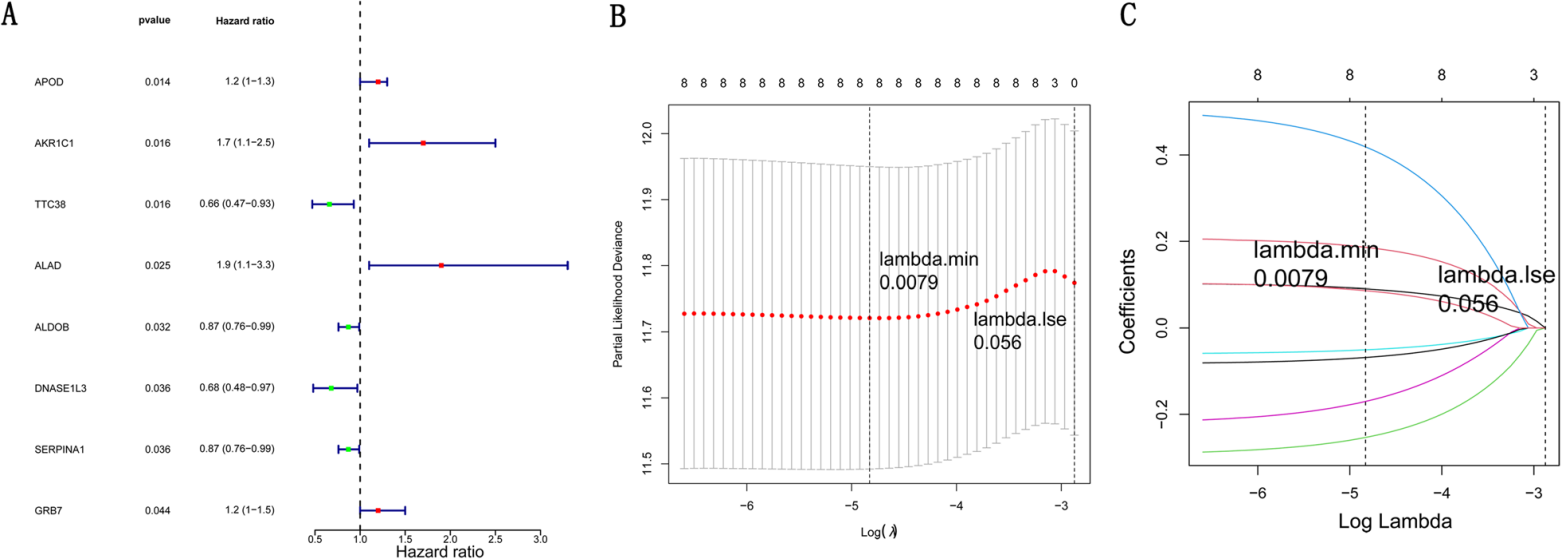
Identification of prognosis-related DEGs. **A** Univariate Cox regression analysis of eight prognosis-related DEGs. **B** LASSO Cox regression analysis eight prognosis-related DEGs. **C** Tenfold cross-validation of eight prognosis-related DEGs

To screen DEGs related to the survival of CRC patients, univariate Cox regression analysis of 112 DEGs were performed in training datasets. As shown in Fig. 3A, we obtained eight prognosis-related DEGs: APOD, AKR1C1, TTC38, ALAD, ALDOB, DNASE1L3, SERPINA1 and GRB7. The eight prognosis-related DEGs were subjected to LASSO Cox regression analysis and tenfold cross-validation to identify the DEGs significantly associated with CRC prognosis. We found that eight DEGs were significantly related to the prognosis of CRC patients at the best lambda value equal to 0.0079 (Fig. 3B, C). Thus, Risk score = 0.0904 × APOD + 0.1861 × AKR1C1 + (− 0.2536) × TTC38 + 0.4189 × ALAD + (− 0.0505) × ALDOB + (− 0.1702) × DNASE1L3 + (− 0.0683) × SERPINA1 + 0.0865 × GRB7.

**Fig. 4:**
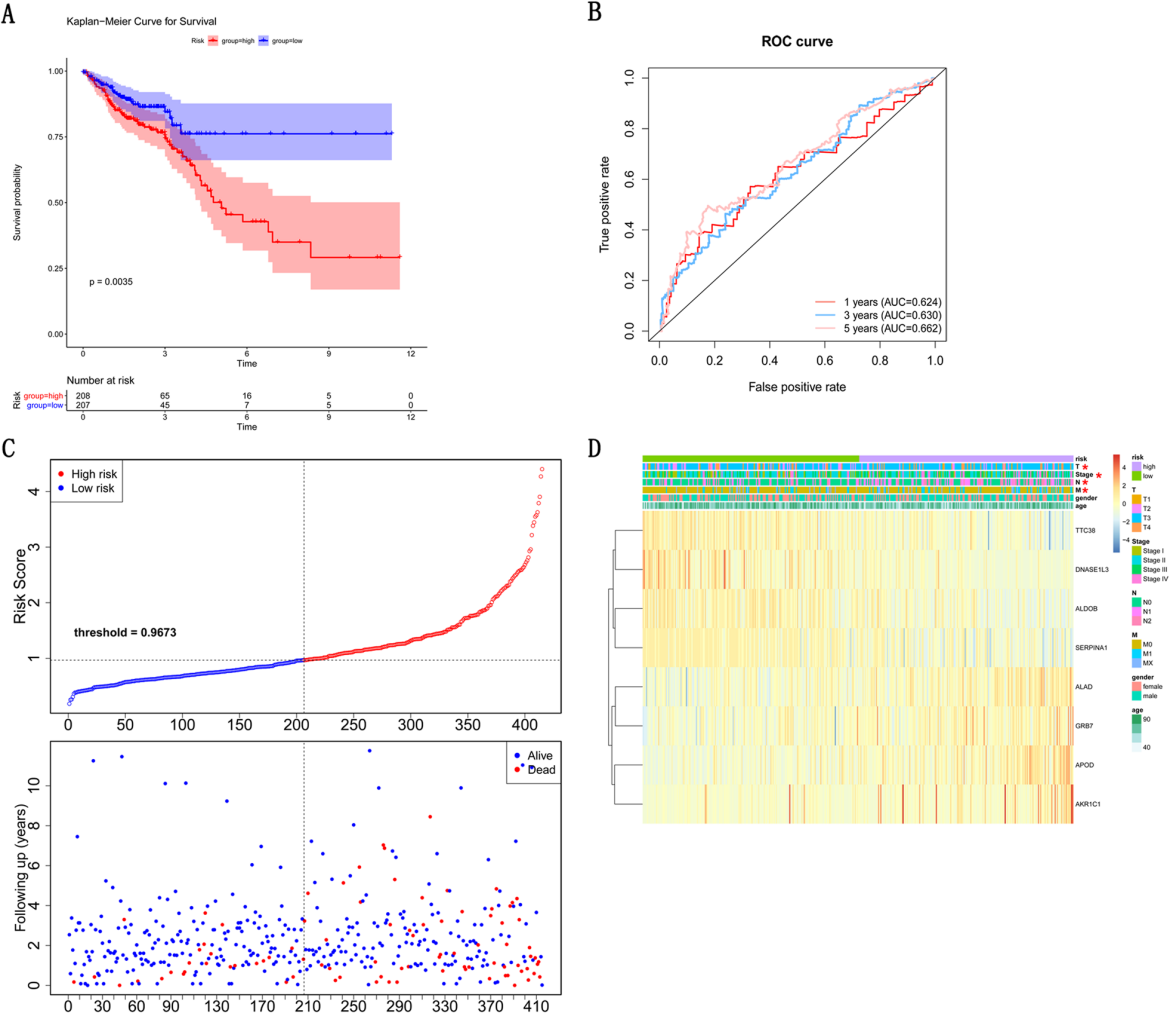
Establishment of eight gene prognostic model in TCGA testing set. **A** The Kaplan–Meier Curve for Survival between high-risk and low-risk patients in TCGA training set. **B** The AUCs of ROC curve analysis in training set. **C** Distribution of the risk curve and survival status between high-and low-risk group in training set. **D** The correlations between eight prognoses related DEGs and clinical characteristics in training set

By using K-M survival curves to assess survival differences between high- and low-risk patients in risk model, the results showed that over time, the survival rate of the high-risk group decreased more significantly than the low-risk group and that the average prognosis was poorer in the high-risk group (*p* = 0.0035) (Fig. 4A). The prognostic value of the K–M survival curve was identified by the ROC curve, and the result found the AUCs of ROC analysis at 1, 3 and 5 years were 0.624, 0.630 and 0.662 (Fig. 4B), respectively. It is indicated that the K–M survival curve has moderate confidence. Figure 4C shows the distribution of risk scores in patients with CRC and the relationship between risk scores and survival time. Furthermore, we analyzed the relationship between eight prognostic-related DEGs with clinical characteristics in the training dataset. As shown in Fig. 4D and Table 1, M stage, N stage, T stage and Stage are significantly correlated with the level of risk score (*p* < 0.05).

**Table 1:** The correlation analysis between eight prognostic-related DEGs with clinical characteristics in the training dataset

	Total	Expression		*p*-value
		High	Low	
	(N = 388)	(N = 193)	(N = 195)	
*Gender*				
female	177 (45.6%)	81 (42.0%)	96 (49.2%)	0.182
male	211 (54.4%)	112 (58.0%)	99 (50.8%)	
*Age (years)*				
> = 60	279 (71.9%)	135 (69.9%)	144 (73.8%)	0.459
< 60	109 (28.1%)	58 (30.1%)	51 (26.2%)	
*M*				
M0	292 (75.3%)	137 (71.0%)	155 (79.5%)	0.034
M1	58 (14.9%)	38 (19.7%)	20 (10.3%)	
MX	38 (9.8%)	18 (9.3%)	20 (10.3%)	
*N*				
N0	224 (57.7%)	88 (45.6%)	136 (69.7%)	< 0.001
N1	93 (24.0%)	57 (29.5%)	36 (18.5%)	
N2	71 (18.3%)	48 (24.9%)	23 (11.8%)	
*T*				
T1	11 (2.8%)	4 (2.1%)	7 (3.6%)	0.003
T2	60 (15.5%)	18 (9.3%)	42 (21.5%)	
T3	274 (70.6%)	144 (74.6%)	130 (66.7%)	
T4	43 (11.1%)	27 (14.0%)	16 (8.2%)	
*Stage*				
Stage I	59 (15.2%)	16 (8.3%)	43 (22.1%)	< 0.001
Stage II	159 (41.0%)	70 (36.3%)	89 (45.6%)	
Stage III	111 (28.6%)	69 (35.8%)	42 (21.5%)	
Stage IV	59 (15.2%)	38 (19.7%)	21 (10.8%)	

### Validation of eight gene prognostic model

**Fig. 5:**
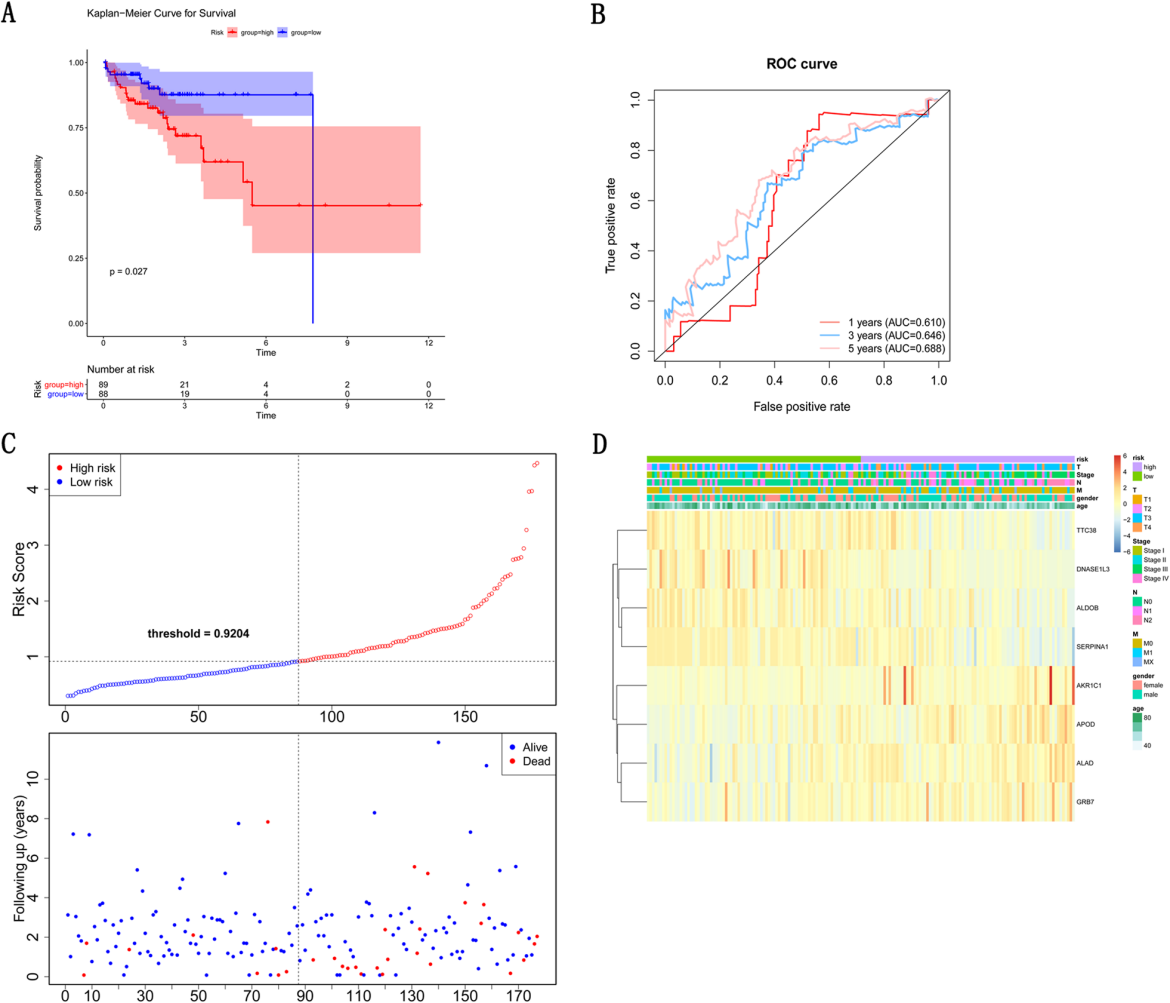
Validation of eight gene prognostic model. **A** The Kaplan–Meier Curve for Survival between high-and low-risk patientsin the TCGA testing set. **B** The AUCs of prognostic model by ROC curve in testing set. **C** Distribution of the risk curve and survival status between high-and low-risk group in test set. **D** The correlations between eight gene expression and clinical features in testing set

**Fig. 6:**
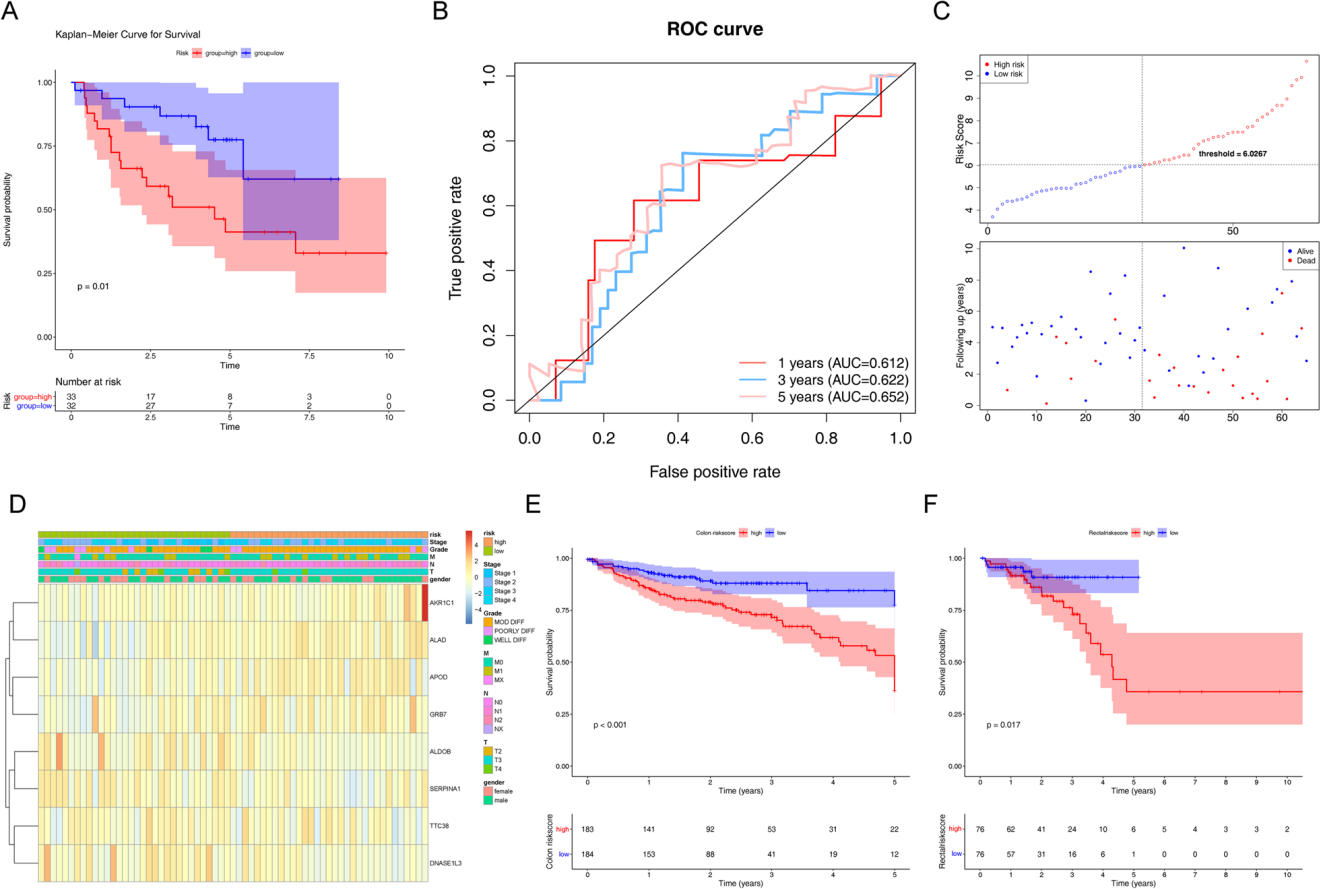
Validation of eight gene prognostic model in GSE29621 set. **A** The Kaplan–Meier Curve for Survival between high-and low-risk patients in GSE29621 set. **B** The AUCs of prognostic model by ROC curve in GSE29621 set. **C** The risk curve and survival status between high-and low-risk group in GSE29621 set. **D** Distribution of the correlations between eight gene expression and clinical features in GSE29621 set. **E** The survival probability between high-and low-riskcore in colon patients. **F** The survival probability between high-and low-riskcore in rectal patients

To determine whether this clinical prognostic model is reliable when applied to different populations, we used the same constructs to evaluate the testing set (TCGA) and validation set (GSE29621). The TCGA testing set includes 177 samples, the GSE29621 includes 65 samples. As shown in Figs. 5A and 6A, high-risk group has shorter survival probability than low-risk group. Due to the limitation of sample size, we further constructed K–M analysis of 5-years OS. The accuracy of prognostic model was evaluated, AUCs of the 1, 3, 5 years ROC curve in testing set were 0.610, 0.646, 0.688, respectively (Fig. 5B). The AUCs of the 1, 3 and 5 years ROC curve in validation set were 0.612, 0.622, 0.652 (Fig. 6B). Furthermore, the patients in testing set and validation set were divided into low-risk group and high-risk group, as shown in Figs. 5C and 6C, with blue dots indicating low-risk patients and red dots indicating high-risk patients, hazardous genes mainly expressed in high-risk group and protective genes mainly expressed in low-risk group. In contrast, there was no significantly correlated between the expression of eight gene signatures and clinical features (T stage, N stage, M stage, stage, gender, and age) in testing set (Fig. 5D and Table 2) and validation set (Fig. 6D and Table 3). These results suggest that clinical prognostic models can accurately predict the prognosis of CRC patients, while some relevant clinical features need to be identified. Meanwhile, we obtained CRC patients’ with survival data from the GSE12945 set to validate the prognostic model. The result found that the AUC scores of ROC curves at 1-, 3-, and 5-year were 0.732, 0.612 and 0.602, respectively, indicating that the prognostic signature had a good predictive performance (Additional file 3: Figure S1). Subsequently, by using K–M survival curves to assess survival differences between high- and low-risk groups of colon cancer patients and rectal cancer patients, the results showed that over time, the survival rate of the high-risk group decreased more significantly than the low-risk group both in colon cancer patients (*p* < 0.001) and rectal cancer patients (*p* = 0.017) (Figs. 6E, F).

**Table 2:** The correlation analysis between eight prognostic-related DEGs with clinical characteristics in the testing dataset

	Total	Expression		*p*-value
		High	Low	
	(N = 170)	(N = 85)	(N = 85)	
*Gender*				
Female	79 (46.5%)	39 (45.9%)	40 (47.1%)	1
Male	91 (53.5%)	46 (54.1%)	45 (52.9%)	
*Age (years)*				
> = 60	119 (70.0%)	52 (61.2%)	67 (78.8%)	0.019
< 60	51 (30.0%)	33 (38.8%)	18 (21.2%)	
*M*				
M0	137 (80.6%)	63 (74.1%)	74 (87.1%)	0.046
M1	23 (13.5%)	17 (20.0%)	6 (7.1%)	
MX	10 (5.9%)	5 (5.9%)	5 (5.9%)	
*N*				
N0	94 (55.3%)	34 (40.0%)	60 (70.6%)	< 0.001
N1	44 (25.9%)	30 (35.3%)	14 (16.5%)	
N2	32 (18.8%)	21 (24.7%)	11 (12.9%)	
*T*				
T1	6 (3.5%)	0 (0%)	6 (7.1%)	< 0.001
T2	38 (22.4%)	11 (12.9%)	27 (31.8%)	
T3	110 (64.7%)	60 (70.6%)	50 (58.8%)	
T4	16 (9.4%)	14 (16.5%)	2 (2.4%)	
*Stage*				
Stage I	40 (23.5%)	10 (11.8%)	30 (35.3%)	< 0.001
Stage II	51 (30.0%)	22 (25.9%)	29 (34.1%)	
Stage III	55 (32.4%)	35 (41.2%)	20 (23.5%)	
Stage IV	24 (14.1%)	18 (21.2%)	6 (7.1%)

**Table 3:** The correlation analysis between eight prognostic-related DEGs with clinical characteristics in the GSE29621 dataset

	Total	Expression		*p*-value
		High	Low	
	(N = 65)	(N = 33)	(N = 32)	
*Gender*				
Female	25 (38.5%)	11 (33.3%)	14 (43.8%)	0.543
Male	40 (61.5%)	22 (66.7%)	18 (56.2%)	
*Grade*				
Mod diff	51 (78.5%)	29 (87.9%)	22 (68.8%)	0.069
Poorly diff	10 (15.4%)	4 (12.1%)	6 (18.8%)	
Well diff	4 (6.2%)	0 (0%)	4 (12.5%)	
*M*				
M0	46 (70.8%)	22 (66.7%)	24 (75.0%)	0.375
M1	18 (27.7%)	11 (33.3%)	7 (21.9%)	
MX	1 (1.5%)	0 (0%)	1 (3.1%)	
*N*				
N0	32 (49.2%)	13 (39.4%)	19 (59.4%)	0.139
N1	25 (38.5%)	17 (51.5%)	8 (25.0%)	
N2	7 (10.8%)	3 (9.1%)	4 (12.5%)	
NX	1 (1.5%)	0 (0%)	1 (3.1%)	
*T*				
T2	8 (12.3%)	0 (0%)	8 (25.0%)	0.006
T3	52 (80.0%)	31 (93.9%)	21 (65.6%)	
T4	5 (7.7%)	2 (6.1%)	3 (9.4%)	
*Stage*				
Stage 1	7 (10.8%)	0 (0%)	7 (21.9%)	0.033
Stage 2	22 (33.8%)	11 (33.3%)	11 (34.4%)	
Stage 3	18 (27.7%)	11 (33.3%)	7 (21.9%)	
Stage 4	18 (27.7%)	11 (33.3%)	7 (21.9%)	

### Univariate and multivariate analyses of independent prognostic factors

**Fig. 7:**
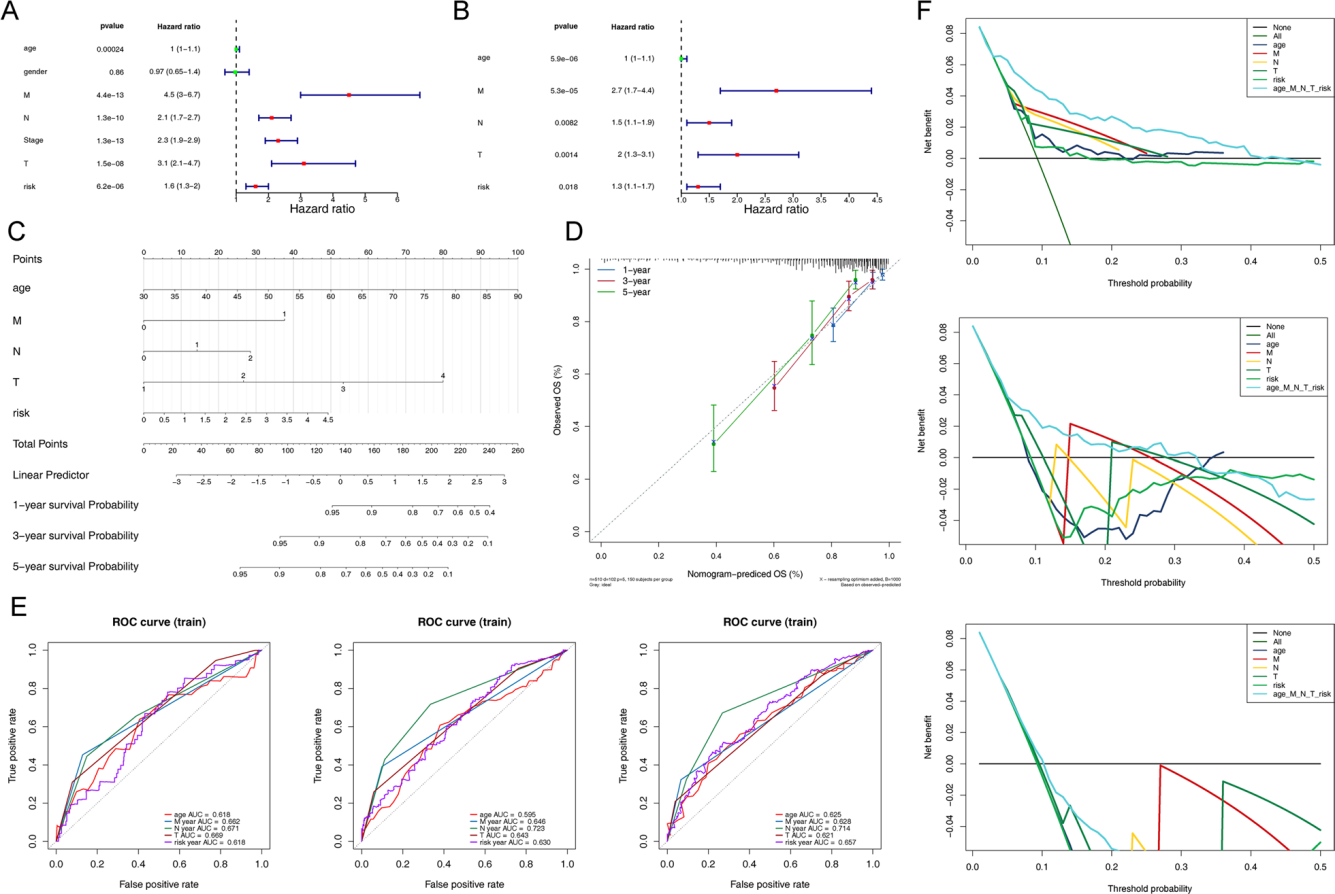
Construction and verification of nomogram based on the TCGA training and testing sets. **A** Univariate Cox regression analysis of independent prognostic factors of CRC patients. **B** Multifactorial Cox regression analysis of independent prognostic factors of CRC patients. **C** Construction of nomogram based on the TCGA training and testing sets. **D** Calibration curve of the nomogram. **E** ROC analysis for prognostic accuracy of independent prognostic factors for CRC in TCGA training set at 1-, 3-, 5 years. **F** Decision curve analysis (DCA) for clinical utilize of nomogram at 1-, 3-, 5 years

To analyze which clinical characteristics are independent prognostic factors affecting patient survival, we performed univariate Cox regression analysis and multivariate Cox regression analysis. Results of univariate Cox regression analysis showed that staging (HR 2.343; 95% CI 1.870–2.935; *p* < 0.001), M stage (HR 4.473; 95% CI 2.982–6.710; *p* < 0.001), N stage (HR 2.120; 95% CI 1.686–2.665; *p* < 0.001), T stage (HR 3.133; 95% CI 2.110–4.651; *p* < 0.001), risk score (HR 1.634; 95% CI 1.321–2.022; *p* < 0.001) and age (HR 1.036; 95% CI 1.017–1.056; *p* < 0.001)were associated with poorer prognosis of CRC patients (Fig. 7A and Table 4). Results of multivariate Cox regression analysis showed that age (HR 1.045; 95% CI 1.025–1.065; *p* < 0.001), M stage (HR 2.694; 95% CI 1.666–4.356; *p* < 0.001), N analysis (HR 1.455; 95% CI 1.456–1.102; *p* < 0.001), T-stage (HR 2.108; 95% CI 1.311–3.104; *p* < 0.001) and risk score (HR 1.334; 95% CI 1.051–1.693; *p* < 0.001) could independently influence prognosis of CRC patients (Fig. 7B and Table 5). These results suggest that our risk score is reasonable as an independent prognostic factor for CRC patients.

**Table 4:** Univariate Cox regression analysis of independent prognostic factors of CRC patients

Variable	Coef	HR	HR.95L	HR.95H	p value
Stage	0.851424618	2.34298233	1.870486654	2.934833128	0.000000000000127
M	1.498098335	4.473174498	2.982146765	6.709693275	0.000000000000443
N	0.751346183	2.119851805	1.68601042	2.665328531	0.000000000127
T	1.142054939	3.13320029	2.11056009	4.651345443	0.0000000147
Risk	0.490970447	1.633901065	1.320612836	2.021510482	0.00000617
Age	0.035417926	1.036052611	1.016627896	1.055848475	0.000244733
Gender	− 0.035011578	0.965594236	0.654021278	1.425599229	0.86019078

**Table 5:** Multivariate Cox regression analysis of independent prognostic factors of CRC patients

Variable	Coef	HR	HR.95L	HR.95H	p value
Age	0.043893632	1.044871208	1.025209238	1.064910264	0.00000594
M	0.991100871	2.694198806	1.666424582	4.355857017	0.0000527
N	0.37557126	1.45582283	1.102169146	1.922953586	0.008166814
T	0.702146062	2.018078987	1.311857686	3.10448522	0.001397312
Risk	0.288178927	1.333995971	1.050954527	1.693265696	0.01786439

### Construction and verification of nomogram

To develop a clinically applicable tool for prognostic assessment of the CRC patients, we built a nomogram based on the clinicopathological features included in the nomogram and extracted from the TCGA training and testing cohorts, including age, M-stage, N-stage, T-stage, and risk score (Fig. 7C). Calibration curves were plotted to assess the accuracy of the predictions of the column line graph with the C-index value is 0.783, and the corrected C-index value is 0.772, indicating the high consistencies between the predicted and observed survival probability. To go a step further, the predicted results of the 1-year prognosis in the nomogram (dashed line) were very close to the actual results (red line), and this prognostic model had a better predictive value for short-term survival (1 year) than long-term survival (2 or 3 years) of the patients. (Fig. 7D). Next, the results of ROC analysis for the survival prediction at 1-, 3, 5 years were exhibited in Fig. 7E, supporting that among the independent prognostic factors for CRC patients had good accuracy with the AUC values greater than 0.6. And meanwhile, the DCA results revealed that the prognostic utilize of nomogram were more excellent compared with individuals (Fig. 7F).

### Molecular characteristics of the high- and low-risk groups

**Fig. 8:**
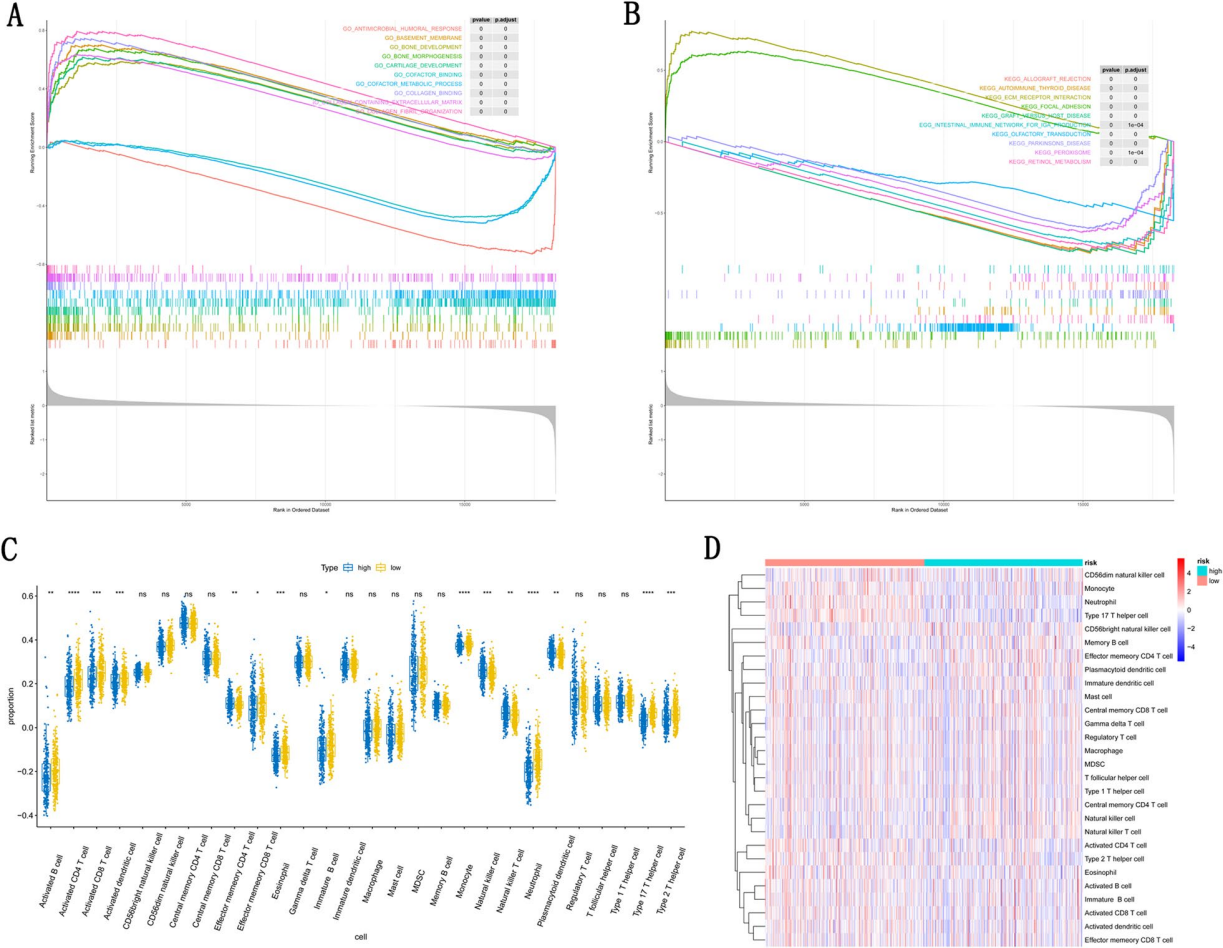
Prognosis gene related functional annotation based on the GSEA and TCGA database. **A** Enriched GO terms between high- and low-risk group in GSEA. **B** Enriched KEGG pathways between high- and low-risk group in GSEA. **C** and **D** Enriched immune cells between high- and low-risk group in TCGA

GSEA was performed to identify significant changes in potential GO terms and KEGG pathways between high- and low-risk populations. The results showed that GO terms such as antimicrobial humoral response, basement membrane, bone development, bone morphogenesis, cartilage development, cofactor binding, cofactor metabolic process, collagen binding and collagen containing extracellular matrix were significantly enriched in the high-risk group (Fig. 8A). Furthermore, allograft rejection pathway, autoimmune thyroid disease pathway, ecm receptor interaction pathway, focal adhesion pathway, graft versus host disease pathway, intestinal immune network for IGA production pathway, olfactory transduction pathway, parkinsons disease pathway, peroxisome pathway and retinol metabolism pathway were significantly enriched in the high-risk group (Fig. 8B). To explore the tumor microenvironment of the disease, we estimated the enrichment of immune cells in different risk tissues in the TCGA dataset. We estimated 28 immune cell subpopulations by using the ssGSEA strategy and showed that patients with CRC in the low-risk group had a relatively high immune status compared to those in the high-risk group (Fig. 8C), and the content of 15 of the 28 cell types was significantly different between the high and low risk groups (Fig. 8D).

### Construction of ceRNA network

**Fig. 9:**
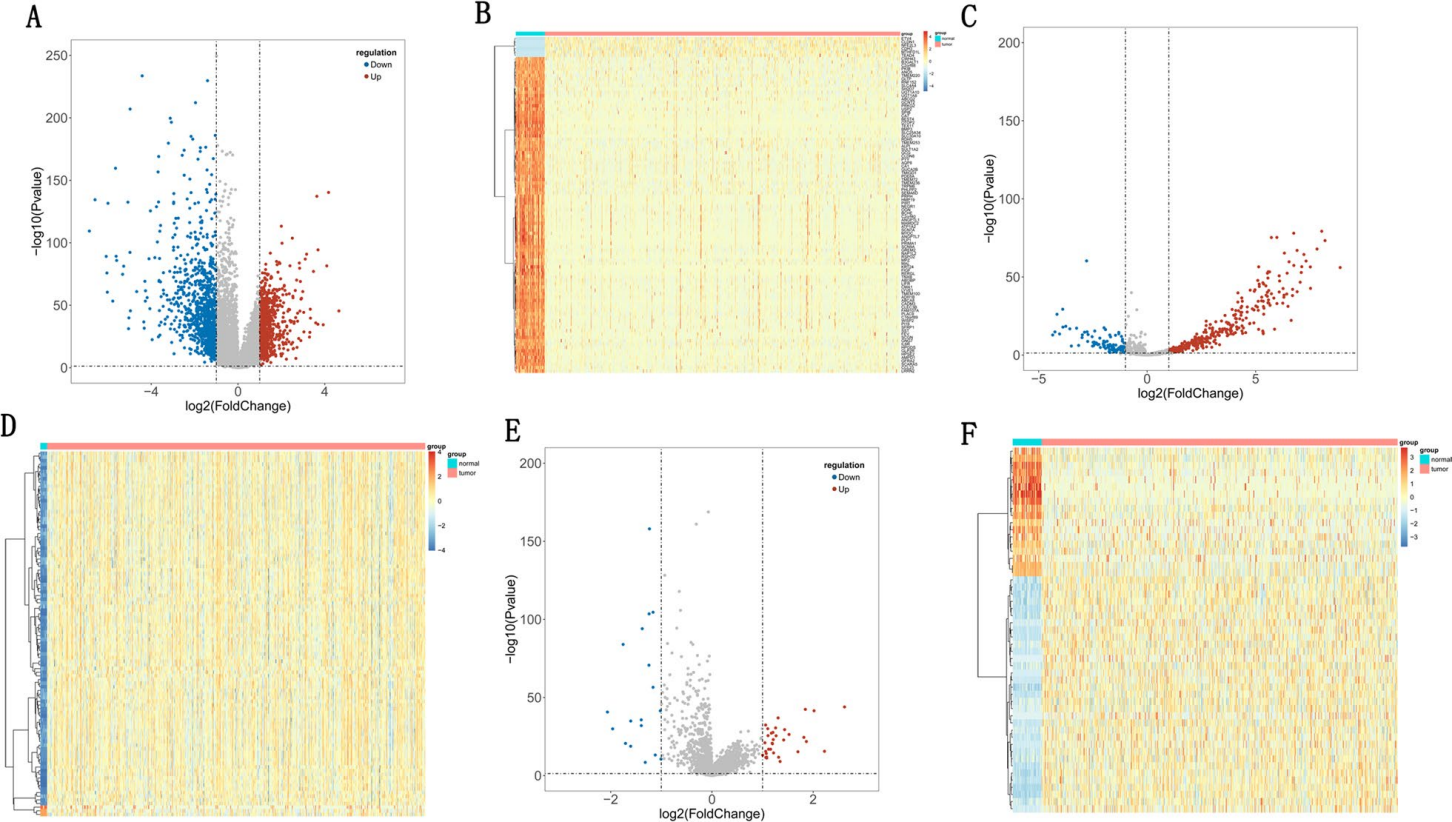
Expression of DEGs, DElncRNAs, and DEmiRNAs between CRC and normal samples in TCGA. **A** A total of 1737 DEGs in TCGA. **B** Heatmap of top 100 DEGs between CRC and CRLM in TCGA. **C** and **D** A total of 462 DEmiRNAs in TCGA. **E** and **F** A total of 51 DElncRNAs in TCGA

**Fig. 10:**
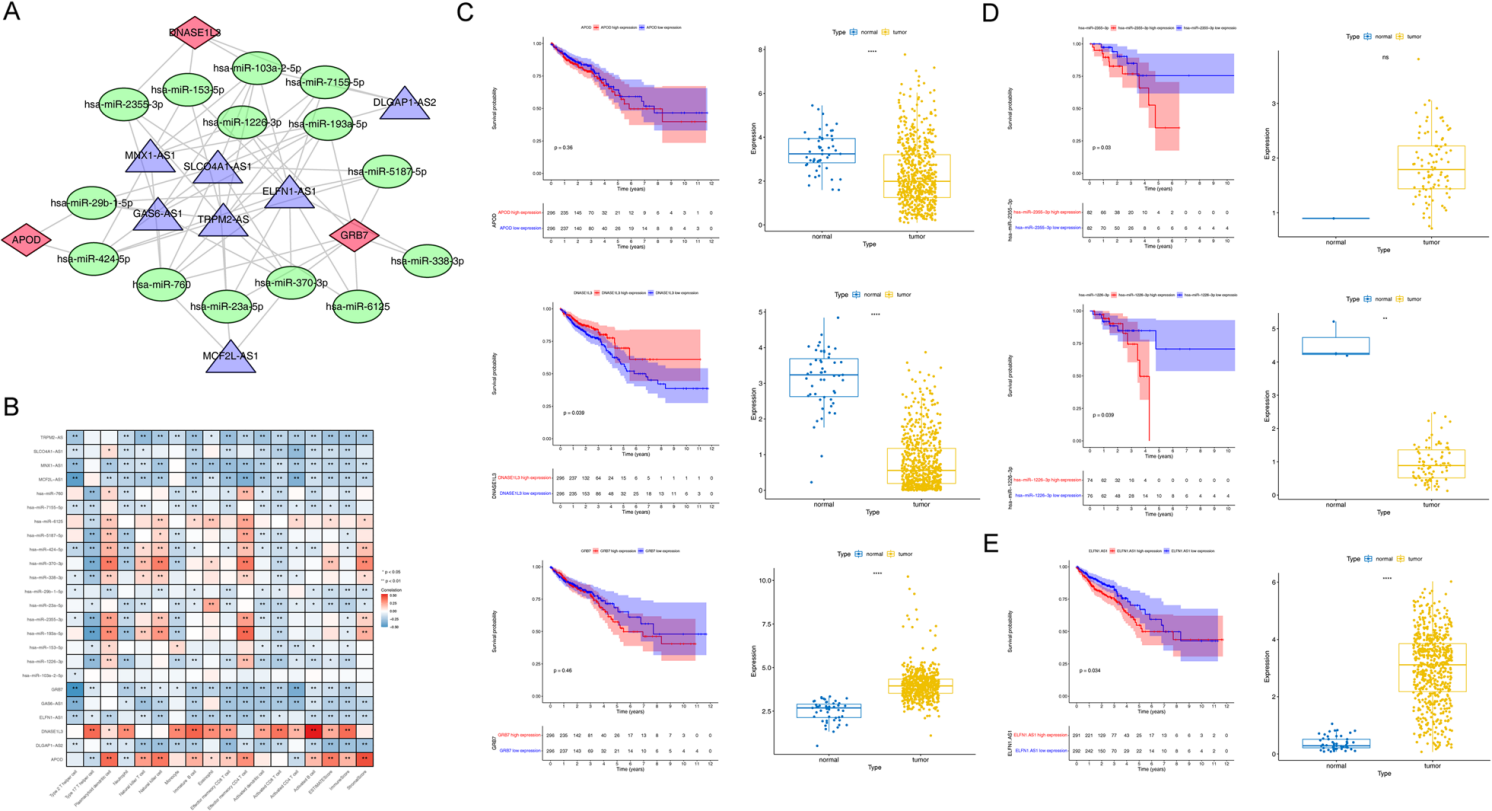
Construction of ceRNA network and validation the accuracy of eight genes model. **A** Construction of ceRNA network. **B** Spearman correlations of key gene expressions with immune cells infiltration. **C** Survival analysis and gene expression of 3 key prognostic genes (DNASE1L3, APOD and GRB7) in the TCGA cohort. **D** Survival analysis and gene expression of key miRNAs (hsa-miR-2355-3p and hsa-miR-1226-3p) in the TCGA cohort. **E** Survival analysis and gene expression of key lncRNA (ELFN1-AS1) in the TCGA cohort. *p < 0.05, **p < 0.01, ***p < 0.001, ****p < 0.0001

We next aimed to investigate DEGs, DElncRNAs, and DEmiRNAs between CRC and normal samples from TCGA cohort. A total of 1737 DEGs were detected, including 780 up-regulated and 957 down-regulated DEGs (Fig. 9A), and the heatmap of top 100 DEGs between CRC and CRLM were shown in Fig. 9B. Similarly, a total of 462 DEmiRNAs were detected, including 335 up-regulated and 107 down-regulated DEmiRNAs (Fig. 9C, D); a total of 51 DElncRNAs were detected, including 33 up-regulated and 18 down-regulated DElncRNAs (Fig. 9E, F). First, we intersected the eight prognostic genes with the DEGs in TCGA and obtained 3 key prognostic genes (APOD, DNASE1L3, GRB7). In addition, the intersection of DEmiRNAs and predicted target miRNA of 3 key prognostic gene were regard as key miRNA. Similarly, the intersection of DElncRNAs and predicted target lncRNAs of key miRNA were regard as key lncRNAs. Finally, we obtained 3 prognosis genes, 14 miRNAs and 7 lncRNAs, which were used to construct ceRNA network (Fig. 10A). Spearman correlation analysis was first used to exhibit the relationship between RNAs involved in the network and the 28 immune factors. As shown in Fig. 10B, these key genes were positively correlated with both immune genes. Next, the survival analysis of key genes, key lncRNAs and key miRNAs were performed in the TCGA cohort. As the results of Fig. 10C, only the individuals with different expression levels of DNASE1L3 had distinct differences in the survival probabilities rether than APOD and GRB7. And meanwhile, the K-M survival curves of the key miRNAs and lncRNAs with significantly differences were displayed in Fig. 10D–E, indicating the cohorts with high expression levels of hsa-miR-2355-3p (*p* = 0.03) and ELFN1-AS1 (*p* = 0.034) had poorer prognosis. While there was greater survival probability in the follow-in case samples with the high expression levels of hsa-miR-1226-3p at 1–2 years. Simultaneously, the gene expression results were consistant with that in survival analysis.

**Fig. 11:**
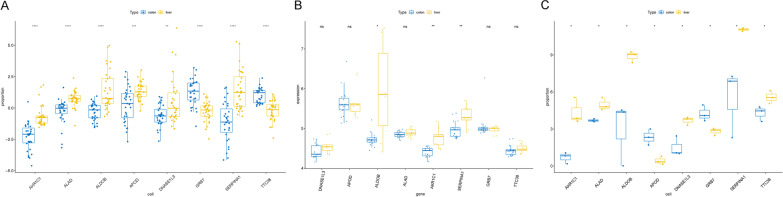
**A** Boxplot of eight prognosis genes expression between CRC and CRLM samples in GSE72718. **B** The expression levels of eight prognosis genes between CRC and CRLM samples in sequencing data. **C** The expression levels of eight prognosis genes between CRC and CRLM samples in GSE22834 dataset. *p < 0.05, **p < 0.01, ***p < 0.001, ****p < 0.0001

To further validate the accuracy of eight prognosis genes to predicted CRLM, the expression of eight prognosis genes was detected in the GSE72718. As shown in Fig. 11A, the expression of the ALDOB, AKR1C1 and SERPINA1 in CRLM samples were significantly up-regulated compared with CRC samples, similar expression trends were obtained in the sequencing data and GSE22834 dataset (Fig. 11B, C). ALDOB, AKR1C1 and SERPINA1 expression were up-regulated in CRC with LM compared to CRC without LM.

**Fig. 12:**
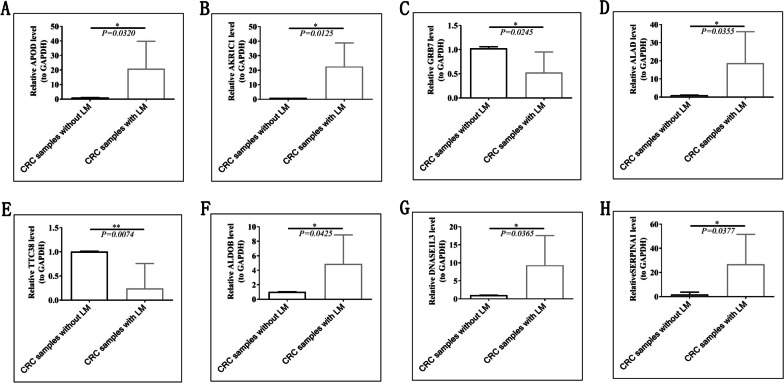
Examination of the expression of eight prognosis-related genes in CRC samples with LM and without LM by qRT-PCR. **A** APOD, **B** AKR1C1, **C** GRB7, **D** ALAD, **E** TTC38, **F** ALDOB, **G** DNASE1L3, **H** SERPINA1. *p < 0.05, **p < 0.01, ***p < 0.001

To further investigate the expression of eight prognosis-related genes in tumor tissues, we performed real-time qPCR using 7 CRC samples with LM and 7 CRC samples without LM. The result showed that APOD, AKR1C1, ALAD, ALDOB, DNASE1L3 and SERPINA1 were high expression in CRC samples with LM, while TTC38 and GRB7 were high expression in CRC samples without LM (Fig. 12).

## Discussion

In this study, the differentially expressed analysis was performed between CRC and CRLM samples. The prognostic model containing eight differential genes were further constructed by univariate Cox regression analysis and LASSO Cox analysis for CRLM identification, that is, the case individuals with CRLM might had poorer prognosis. The prognostic value and clinical utilize of the risk model was varified based on the CRC-related datasets.

Among eight prognosis-related genes, APOD was considered as a good diagnostic marker for CRC [23]. DNASE1L3 might be a biomarker associated with prognosis and immune infiltration in CRC [24]. Another study showed that miR374a-5p could promote metastasis of CRC by targeting GRB7 [25]. These results proved that the model contributed to judging the prognosis of CRC patients.

Functional analysis indicated that antimicrobial humoral response and ECM receptor interaction pathway were significantly enriched in high-risk group. One study found that antimicrobial interventions could reduce Fusobacterium load, cancer cell proliferation, and tumor growth and metastasis in vivo [26]. Another study suggested that the gut microbiome depletion by oral antibiotics inhibited the growth and liver metastases of CRC in murine model [27]. Yuzhalin AE [28] et al. showed that CRLM growth depends on PAD4-driven citrullination of the extracellular matrix. ECM proteins were supposed to act as candidate serological or tissue biomarkers and potential targets for imaging of occult metastases and residual or recurrent tumors [29]. Therefore, we speculate that antimicrobial human response and ECM receiver interaction pathway might be involved in CRLM progression. It should be noted that AUC greater than 0.7 is considered to have high accuracy of diagnostic model, while AUC greater than 0.6 is considered to have high accuracy of prognostic model. Several studies also showed that the accuracy of the prediction model is good based on AUC > 0.6 [30, 31]. The area under the ROC curve of 1-5-year overall survival predicted in our study is greater than 0.6, indicating that the accuracy of the prediction model is good.

The abnormal enrichment of immune cells in the TME was a significant sigh in formation of the premetastatic niche. The ssGSEA showed that the content of 15 among 28 cell types was significantly different between high- and low-risk groups. The interaction between tumor and tumor-associated macrophages (TAMs) in TME of metastasis promote CRLM [32, 33]. TAMs also could enhance the migration, invasion and circulating tumor cell (CTC)-mediated CRLM by inducing EMT [34]. One study showed that neutrophil extracellular traps promote the development and progression of liver metastases after surgical stress [35]. A meta-analysis indicated that an elevated pretreatment neutrophil-to-lymphocyte ratio(NLR) was closely correlated with poor long-term survival (OS and RFS) in CRLM patients [36]. The aggregation of immune cells in TME could exert a significant impact on process of CRLM.

In order to complete the construction of the potential ceRNA network in CRC progression, the key prognostic genes (APOD, DNASE1L3, GRB7) as well as the key prognostic miRNA (hsa-miR-2355-3p, hsa-miR-1226-3p), lncRNA (ELFN1-AS1) were identified by taking the intersection of eight prognostic genes and DEGs, targeted miRNA and DEmiRNA, targeted lncRNA and DElncRNA, respectively. The prognostic value of which were confirmed in the TCGA cohorts as well. Several studies showed that ALDOB-mediated fructose metabolism drives metabolic reprogramming of CRLM [9, 37, 38]. SerpinA1 promoted CRC progression through fibronectin, it might act as a novel prognostic biomarker and candidate therapeutic target for CRC [39]. Lnc MNX1-AS1 could drive proliferation via a MYC/MNX1-AS1/YB1 signaling pathway in CRC [40]. LncRNA DLGAP1-AS1 contributed to CRC progression and 5-FU resistance by regulating smad2 pathway [41]. ELFN1-AS1 accelerated the proliferation and migration of colorectal cancer via regulation of miR-4644/TRIM44 axis [42]. Knockdown of DNASE1L3 would induce the expression of c-Myc protein in HCC cells [43]. MYC-driven up-regulation of lncRNA ELFN1-AS1 could silence TPM1 through epigenetic, and further promote tumor growth of CRC [44]. Overexpression of c-Myc would also increased expression of Serpina1 in metastatic pancreatic cancer, which was consistent with our conclusions. The above results showed that genes, miRNAs and lncRNAs in the ceRNA network demonstrated a strong correlation with the tumorigenesis and progression of CRC.

In this study, construction and validation of the prognostic model for CRLM identification were performed based on the CRC and CRLM cohorts, and meanwhile, the potential ceRNA network targeting the key prognostic genes were predicted for the mechanism exploration in CRC progression. However, there are still some limitations in this paper that the insufficient number of sequencing samples might not fully confirm its effectiveness, which required enlarging clinical samples to complete the verification.

## Conclusions

In summary, the prognostic risk model which contained eight genes was confirmed to possess a high prognostic value and could independently identify high-risk case patients with low survival. The relationships between immune microenvironment and CRC prognosis were evaluated as well. Moreover, the key prognostic genes-related ceRNA network were established for the CRC investigation. We make the case that the study may provide inspiration for further research on the pathogenesis of CRC and CRLM.

## Supplementary Information

**Additional file 1**. **Table S1**: The detailed information of 10 CRC samples with LM and 10 CRC samples without LM involved in the study.

**Additional file 2**. **Table S2**: The primers of quantitative real-time PCR in the study.

**Additional file 3**. **Figure S1**: Evaluation the accuracy of prognostic model in the GSE12945 cohort

## Data Availability

TCGA-COAD and TCGA-READ datasets were downloaded from the TCGA (https://portal.gdc.cancer.gov/) database. GSE22834, GSE29621, GSE12945 and GSE72718 datasets were soured from the GEO (https://www.ncbi.nlm.nih.gov/geo/) database.
