# Supplementary figures and images for: Establishment and verification of prognostic model and ceRNA network analysis for colorectal cancer liver metastasis
Source: BMC Med Genomics. 2023 May 9;16:99. doi: 10.1186/s12920-023-01523-w (PMC10169504; doi:10.1186/s12920-023-01523-w)

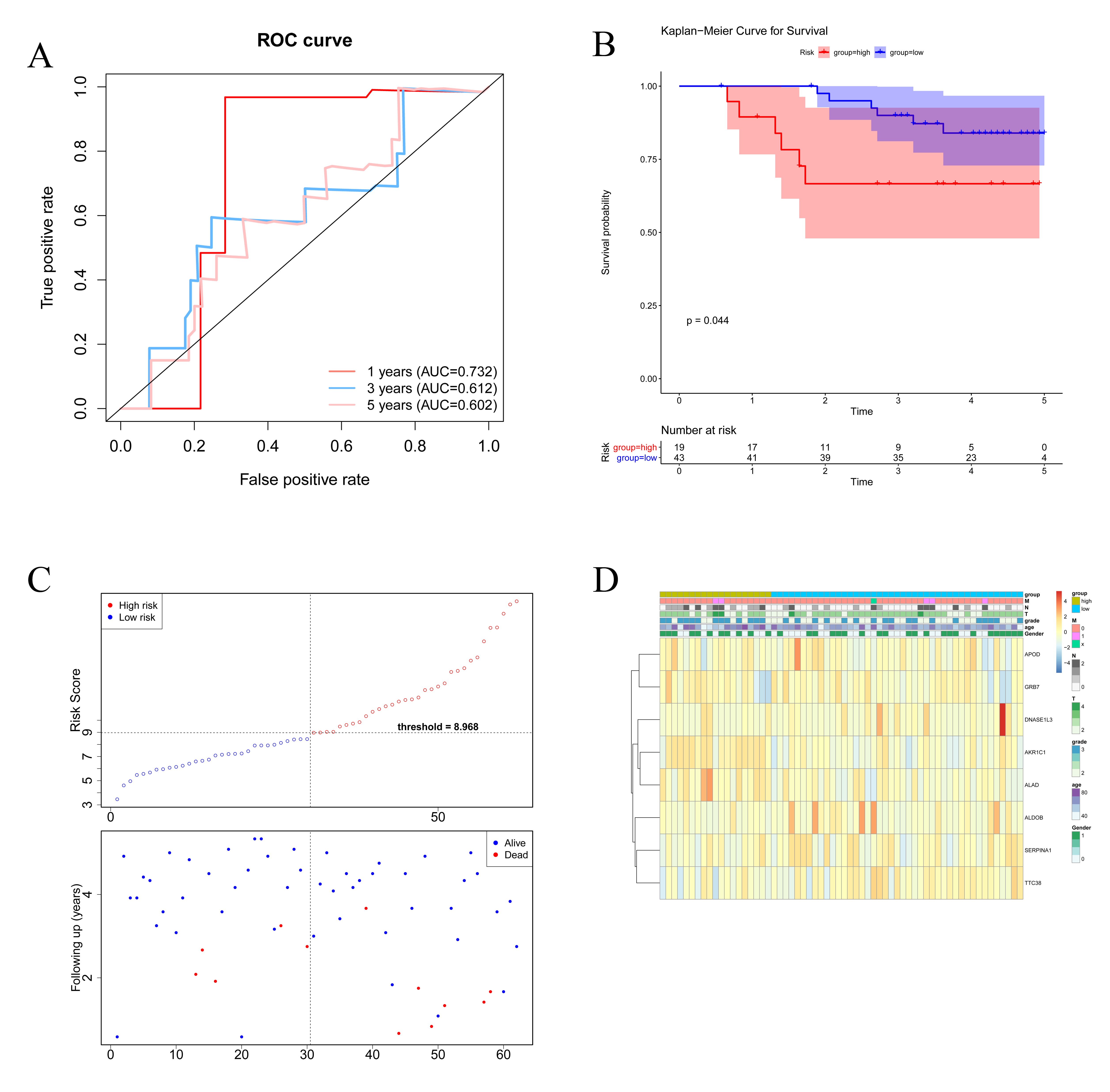

Supplement: Supplementary file 3 — Additional file 3. Figure S1: Evaluation the accuracy of prognostic model in the GSE12945 cohort [file 12920_2023_1523_MOESM3_ESM.tif]
